# Clinical outcome of breast cancer occurring after treatment for Hodgkin's lymphoma: case-control analysis

**DOI:** 10.1186/1748-717X-4-19

**Published:** 2009-06-30

**Authors:** Mohamed A Alm El-Din, Kevin S Hughes, Rita A Raad, Saveli I Goldberg, Alan C Aisenberg, Andrzej Niemierko, Alphonse G Taghian

**Affiliations:** 1 Department of Radiation Oncology, Massachusetts General Hospital, Harvard Medical School, Boston, MA 02114, USA; 2 Department of Surgical Oncology, Massachusetts General Hospital, Harvard Medical School, Boston, MA 02114, USA; 3 Department of Medical Oncology, Massachusetts General Hospital, Harvard Medical School, Boston, MA 02114, USA; 4 Department of Radiation Oncology, Tanta University Hospitals, Tanta Faculty of Medicine, Tanta, Egypt

## Abstract

**Background:**

To evaluate diagnosis, management and outcome of breast cancer (BC) occurring after irradiation for Hodgkin's lymphoma (HL).

**Methods:**

39 cases of BC in 28 HL survivors were retrospectively reviewed. 21 patients were included in a case-control analysis.

**Results:**

The median age at diagnosis of HL and BC was 25.3 and 45.3 years, respectively. The median interval to develop BC was 16.1 years. Eleven women (39.2%) had bilateral disease. Mode of detection of the index breast cancers was by mammographic screening in 17 patients (60.7%), palpable lump in 8 patients (28.6%), clinical examination in two patients (7.1%), and unknown in one patient (3.6%). Case-control analysis showed that histological features and prognosis of BC after HL were similar to those of primary BC, however, for BC after HL, mastectomy was the predominant surgery (*P *= .001) and adjuvant radiotherapy and anthracycline-based chemotherapy were less frequently used as compared to primary BC (*P *< .001 and .003, respectively).

**Conclusion:**

The previous history of HL does not appear to be a poor prognostic factor for BC occurring thereafter.

## Background

The improved survival rates among Hodgkin's lymphoma (HL) patients have brought with it added long-term morbidities. In particular, breast cancer (BC) has been a major concern among women irradiated for HL at a young age [1-7], where the risk of BC is significantly higher 15 years or more after mantle radiation [3-8]. The experience from atomic bomb survivors emphasizes the delayed onset of radiation-induced BC [9]. The history of previous irradiation and chemotherapy (CT) has significant implications on the management of BC among those patients. Furthermore, it is not clear whether the prognosis of BC among this population is worse, better or the same as that of primary BC. This report serves to address issues of diagnosis, management and outcome of BC that occurs after HL.

## Patients and methods

With institutional review board (IRB) approval, we retrospectively reviewed the medical records of twenty-eight women who developed 39 in-situ or invasive breast cancers. These women were treated for HL between 1959 and 1999; twenty-four patients were treated at Massachusetts General Hospital, while 4 patients were treated elsewhere. All were treated for their BC at Massachusetts General Hospital between 1981 and 2005.

The original surgical pathology reports, medical and Tumor Registry records were reviewed. The details of HL treatment were reviewed [treatment modality, radiotherapy (RT) machine, RT dose, RT field, and CT regimens], as well as the mode of presentation of the index breast cancers. Pathological characteristics of breast cancers as well as tumor location within the breast were recorded. We evaluated the pathological type, T-stage, and axillary nodal status of the first tumor in patients who had bilateral disease. Treatment details of breast cancers were also collected including surgical procedure, adjuvant RT and/or systemic treatment.

Hazards estimate for metachronous bilateral BC was calculated as the number of cancers during the follow-up period divided by the total number of women-years at risk in that interval [10]. The median follow-up after the first BC was 63.4 months (range, 8.9 to 301.7 months) with a total of 186 patient years (149 patient years after exclusion of patients with synchronous BC).

To address the treatment as well as the outcome of the index BC occurring after HL as compared to primary BC, we conducted a case-control analysis for patients with invasive tumors. We excluded from our case-control analysis all women with ductal carcinoma in-situ (DCIS) (3 patients), patients where less than 3 matches could be found in our database (2 patients), and patients with some information missing (2 patients). For each patient of the remaining 21 patients, 3 patients with BC and no history of HL were randomly selected from our database. The cases were matched for five criteria: age (within 5 years), year of diagnosis (within 5 years), tumor size, nodal positivity (0, 1 to 3, > 3) and estrogen receptors status (positive versus negative). If the exact match was not available, we relaxed the selection criteria on only four attempting to choose a comparison patient with less favorable prognostic feature (e.g. larger tumor size, etc).

As a result, 21 patients with BC after HL were compared to a group of 63 patients with primary BC. The median follow-up in the 21 patients was 62.3 months (range, 8.9 to 301.7 months) and 71.9 months (range, 3.8 to 292 months) in the control group. For patients with synchronous bilateral disease, we matched the tumor with the worst pathological features and for those with metachronous disease we matched the first BC. Both groups were compared for histological features, treatment, and outcome, including disease-free and overall survival. Exact Fisher's test was used to assess differences between the study group and the comparison group in the distribution of prognostic variables and treatment approaches. Survival curves for study and comparison groups were estimated using the Kaplan-Meier method [11].

## Results

### Treatment for HL

Table 1 details the age distribution of HL and BC diagnoses as well as the interval to develop BC after HL. All patients received RT to lymph node-bearing areas above the diaphragm (Table 2). Twenty-five patients received RT to all lymph nodes areas that are included in a standard mantle field (neck, supraclavicular, infraclavicular, axilla and mediastinum). Two patients had RT to modified mantle field where axillary nodes were not included, and one patient had involved-field RT to the neck and supraclavicular nodes. Two patients also had RT for relapsed disease; one of them received additional dose to the mediastinum and the other received RT to Waldeyer's ring. The median radiation dose delivered to the mediastinum was 39.6 Gy (range, 25.2 to 46.2 Gy). Fourteen patients also had CT, eleven for primary disease and three for relapse. For primary disease, five patients received doxorubicin, bleomycin, vinblastine and dacarbazine (ABVD) (one of these patients received two cycles of etoposide, vinblastine and doxorubicin after four cycles of ABVD), four patients received nitrogen mustard, vincristine, procarbazine and prednisone (MOPP), one patient received MOPP/ABVD (this patient received additional cycle of ifosphamide, carboplatin and etoposide) and one patient received only nitrogen mustard. For relapse, one patient received MOPP, one patient received MOPP/ABVD and the third patient received a combination of chlorambucil and vinblastine.

**Table 1:** Age distribution for Hodgkin's lymphoma, breast cancer occurring thereafter and time interval in-between

**HL**	
Age (years)	No. of patients (%)
11 – 19 20 – 29	9 (32.1) 8 (28.6)
30 – 60	11 (39.3)

**BC**	

Age (years)	No. of patients (%)
≤ 35	7 (25)
36 – 49	11 (39.7)
≥ 50	10 (35.3)

**HL – BC Interval**	

Interval (years)	No. of patients (%)
< 10	3 (10.7)
10 – 20	19 (67.8)
> 20	6 (21.5)

Abbreviations: HL, Hodgkin's lymphoma; BC, breast cancer

**Table 2:** Hodgkin's lymphoma treatment

	**No. of patients**	**%**
**Modality**		
RT only	14	50
RT + CT	14	50
RT machines *		
Van de Graff	8	32
Linear accelerator 4 MV	1	4
Linear accelerator 6 MV	5	16
Linear accelerator 10 MV	12	48
**Dose to mediastinum (Gy) ¶**		
None ‡	1	3.7
20 –40	14	51.8
≥40	12	44.5
**RT field**		
Standard mantle	9	28.6
Modified mantle	2	10.7
TLI/STLI§	16	57.2
IF	1	3.5

Abbreviations: RT, radiotherapy; STLI, subtotal lymphoid irradiation; TLI, total lymphoid irradiation; IF, involved field, CT, chemotherapy.

* Data were not available for two patients.

**¶ **Dose was unknown for one patient.

**‡ **This patient received only involved field RT to left neck and supraclavicular lymph nodes.

§ One of these patients also received unilateral lung irradiation to 15 Gy.

### Breast Cancer: Clinical Information

The median age at diagnosis of the index BC was 45.3 years (range, 22 to 66 years). The median interval to develop BC from treatment of HL was 16.1 years (range, 4 to 36 years) (Table 1). Of the index breast cancers, tumors were detected by mammography in 17 patients (60.7%), breast self-examination in 8 patients (28.6%), clinical examination in two patients (7.1%) and unknown in one patient (3.6%). Eleven patients (39.2%) developed bilateral tumors; one of them developed in-breast recurrence and contralateral invasive carcinoma at the same time, seven years after conservative surgery for DCIS.

Of the eleven contralateral tumors, seven were detected by mammography, one was detected during clinical examination and one was detected by breast-self examination. Two occult contralateral breast cancers were found among six prophylactic mastectomies. Table 3 details the histology of bilateral tumors and time interval in-between. Family history was positive for 3 out of 11 patients with bilateral disease. Only one patient had first-degree relative with history of BC.

**Table 3:** Pathological types of first and contralateral breast cancers and time interval in-between in patients with bilateral disease

**Patient No**	**First**	**Second**	**Interval (months)**
1	DCIS	Invasive	0.0
2	Invasive	DCIS	0.0
3	DCIS	Invasive	1.4
4	Invasive	DCIS	2.8
5	Invasive	Invasive	9.6
6	Invasive	Invasive	42.6
7	Invasive	Invasive	55.1
8	Invasive	Invasive	77.7
9	DCIS	Invasive	83.2
10*	Unknown	DCIS	130.6
11	Invasive	DCIS	197.1

Abbreviations: DCIS, ductal carcinoma in situ.

* Patient was not treated for her first breast cancer at Massachusetts General Hospital.

- Synchronous disease: second breast cancer diagnosed within six months after the first.

- Metachronous disease: second breast cancer occurred more than six months after the first.

The location of breast cancers could be determined in 34 of the 39 cases; 23 (59%) upper outer quadrant, 2 (5.1%) lower outer quadrant, 2 (5.1%) upper inner quadrant, 2 (5.1%) lower inner quadrant, 3 (7.7%) mid-upper, one (2.6%) central, one (2.6%) multiple quadrants and 5 (12.8%) unknown.

### Breast Cancer: Pathology & Stage

Of the 28 index breast cancers, 21 (75%) were infiltrating duct carcinoma (one with mucinous features), one (3.6%) was infiltrating lobular carcinoma, one (3.6%) was infiltrating cancer with both ductal and lobular features, and 4 (14.3%) were DCIS. Pathologic type was unknown for one tumor (3.5%). For invasive tumors, pathologic T-stage was available for 22: 16 (69.6%) were T1, 5 (21.7%) were T2, one (4.3%) was T4 and one (4.3%) was unknown. Seven patients (31.8%) had positive axillary lymph nodes, where 15 patients (68.2%) had negative nodes.

Of the eleven cancers found contralaterally, six tumors were infiltrating duct carcinoma, and five were DCIS. For invasive tumors, four tumors were T1 and two tumors were T2. Axillary lymph nodes were positive for two, negative for two, and unknown for two tumors.

The case-control analysis (Table 4) showed no significant difference regarding the histological features (grade or lymphovascular invasion) of the index breast cancers occurring after HL as compared to those of breast cancers in the control group.

**Table 4:** Case-control analysis

	**Study group** **(21 patients)** **No (%)**	**Control group** **(63 patients)** **No (%)**	**P value**
Menopausal status			
Premenopausal	12 (57.1)	40 (64.5)	0.7
Postmenopausal	9 (42.9)	22 (35.5)	

Grade			0.5
1	3 (14.3)	8 (12.7)	
2	11 (52.4)	24 (38.1)	
3	5 (23.8)	26 (41.3)	
Unknown	2 (9.5)	5 (7.9)	

LVI			0.8
Present	7 (33.3)	16 (25.4)	
Absent	11 (52.4)	37 (58.7)	
Unknown	3 (14.3)	10 (15.9)	

Surgery			
Mastectomy	18 (86)	20 (32)	0.001
Lumpectomy	3 (14)	43 (68)	

Adjuvant RT			
Yes	4 (19.0)	49 (78)	< 0.001
No	16 (76.2)	14 (22)	
Unknown	1 (4.8)		

Adjuvant anthracyclines			
Yes	2 (9.5)	30 (47.6)	0.003
No	18 (85.7)	31 (49.2)	
Unknown	1 (4.8)	2 (3.2)	

Abbreviations: RT, radiotherapy; LVI, lymphovascular invasion

### Breast Cancer: Treatment and Outcome

**Figure 1:**
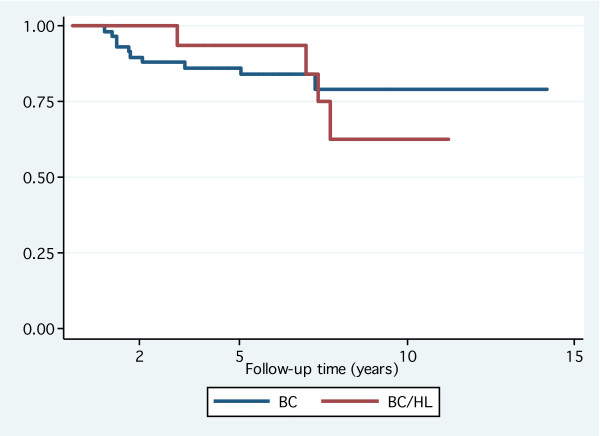
**Disease-free survival**: No significant difference between breast cancer after Hodgkin's lymphoma and primary breast cancer; log-rank test: *P *= 0.9. Abbreviations: BC, breast cancer; HL, Hodgkin's lymphoma.

**Figure 2:**
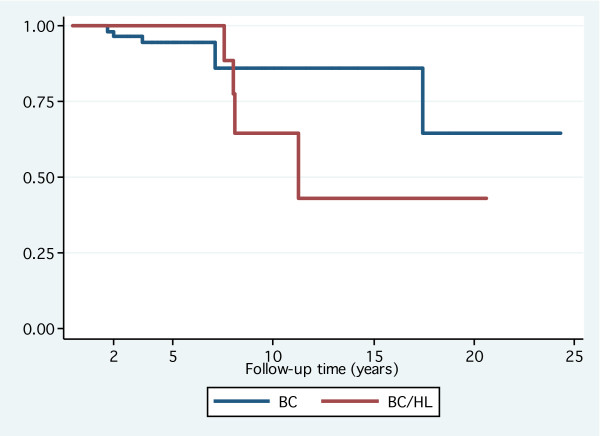
**Overall survival**: No significant difference between breast cancer after Hodgkin's lymphoma and primary breast cancer; log-rank test: *P *= 0.4. Abbreviations: BC, breast cancer; HL, Hodgkin's lymphoma.

Among 21 patients with BC after HL who were included in the case-control analysis, only three patients were treated with lumpectomy while the reminder was treated by mastectomy in light of prior radiotherapy for HL. Two patients felt to be at higher risk for loco-regional failure received adjuvant chest wall RT following mastectomy. These two patients had 6 out of 15 and 3 out of 5 positive axillary lymph nodes, respectively. None of them had experienced any complications from RT at their last follow-up (4.3 and 8 years, respectively). Following lumpectomy, adjuvant RT was declined (as well as adjuvant systemic therapy) by one patient while it was given for the other two. One patient received whole breast RT to a dose of 50 Gy followed by a 10 Gy boost to the tumor bed. The other patient received fractionated partial breast irradiation by 3-dimensional conformal technique (50 Gy in 25 fractions) to the lumpectomy site after refusing mastectomy [12]. The cosmetic results for both patients were reported as excellent 36 and 27 months after RT, respectively. With regards to adjuvant systemic therapy, 13 out of 21 patients received CT and/or hormone therapy with only two patients had anthracycline-based regimens. The case-control analysis highlighted the differences in management between both groups with mastectomy being more frequent (*P *= .001), and consequently adjuvant RT was less frequent (*P *< 0.001) in patients with BC after HL (Table 4). Patients with primary BC received more anthracyclines in their adjuvant treatment compared to patients with BC after HL (*P *< 0.003). The 5 and 10-year disease-free survival in the study group was 94% (95% confidence interval [CI]: 63–99) and 62% (95% CI: 26–85) compared to 84% (95% CI: 74–93) and 79% (95% CI: 62–89) in the control group, respectively. The 5 and 10-year overall survival in the study group was 100% and 65% (95% CI: 25–87) compared to 95% (95% CI: 84–98) and 86% (95% CI: 67–94) in the control group, respectively. Overall, there was no significant difference in disease-free or overall survival between both groups (Figures 1 and 2, respectively).

## Discussion

The median interval between diagnosis of HL and development of BC was 16.1 years which was similar to the intervals reported in series from Stanford (17 years) [13], Memorial Sloan Kettering Cancer Center (15 years) [10] and Mayo Clinic (19.9 years) [6]. Despite the long interval to develop radiation-induced BC, the median age of BC diagnosis in this cohort was 45.3 years. This concurs with results from other studies reporting that BC after treatment of HL occurs at a relatively younger age compared to primary BC [6,10,13].

The majority of index breast cancers (60.7%) in our series were discovered during screening mammography. This number might be an underestimation since we were not able to determine the date of the last mammogram in individuals presenting with clinically apparent BC. Yahalom et al [10] and Dershaw et al [14] also reported the success of mammography in detecting 80–90% of breast cancers among their cohorts. It is quite possible these studies included patients who were better screened, or more compliant with screening. Dershaw's group compared this technique with physical examination, which revealed fewer than 40% of the tumors. However, breast self-examination or clinical examination was reported by other studies as the prevalent method of detection [3,13,15]. While our study was not designed to evaluate screening, the high rate of Tis and T1 cancers found, and the high rate of mammographically detected cancers in our and other studies, highlights the importance of intensive screening.

In patients with primary BC, the reported incidence of bilateral disease is variable, ranging from 4% to 21%, the majority of cancers being metachronous [16,17]. Eleven of our 28 patients (39.2%) developed bilateral BC; four of whom had synchronous tumors (14.2%) and seven had metachronous tumors (25%). This rate is significantly higher than that reported in the general population and also higher than those reported by Bhatia et al (29%) [1] and Yahalom et al (22%) [10]. Of note, our cohort has longer median follow-up compared to these two reports (5 years versus 3 years for each). Furthermore, it should be noted that women who develop BC at young age are at an increased risk to develop contralateral disease [18] as this may reflect more years of follow-up and smaller risk of death from other causes [19]. In our series, the average annual hazards rate for metachronous bilateral BC (3.2%) was higher than that of primary BC (0.5 to 1%) [20-24] and also higher than that reported in other studies for BC after HL (1.36% to 2.6%) [10,13]. Whether this higher rate of bilaterality warrants surgical prophylaxis is an open question. As second tumors seem to be detected quite early with vigilant mammographic screening, and as Magnetic Resonance Imaging (MRI) screening may allow more complete early detection, the role of prophylaxis remains a personal choice.

The majority of breast cancers (64.1%) in our patients were laterally located within the breast, with the upper outer quadrant being the most frequent location (59%). This concurs with results from other reports [3,6,13,25,26] and is also similar to the incidence of upper quadrant tumors in primary BC (61–65%) [27,28]. In a study of doses delivered to the breast during mantle irradiation, unshielded upper outer quadrant appears to receive higher radiation doses compared to tissue beneath the lung block [29]. Of interest, some authors have reported a higher incidence of medially located tumors for patients who develop BC after HL [1,10,15]. Apparently, radiation-induced breast cancers following treatment for HL may occur anywhere in the breast. This might be inferred from the study reporting large dose gradient (3–42 Gy) across the breast following typical mantle treatment with a midline dose of 40 Gy [30]. There is convincing evidence for a strongly linear radiation dose response in the lower dose range (up to 5 or 10 Gy) [31-36]. Therefore, low doses of radiation delivered incidentally to any of the breast quadrants appear to be of concern. This was also confirmed in the setting of RT for BC; Stovall et al reported that women < 40 years of age who received > 1 Gy of absorbed dose to the specific quadrant of the contralateral breast had a 2.5-fold greater risk for contralateral BC than unexposed women [37].

We evaluated the pathological type, T-stage, and axillary nodal status of the index BC in patients who had bilateral disease, as the contralateral tumors are often detected at an early stage due to intensive screening. The incidence of axillary nodes involvement in our series was 31.8%, which was similar to the 31% [10] and 27% [13] reported by others, and also similar to T-stage adjusted rate in primary BC [38]. On the other hand, Cutuli al al [39] reported higher incidence of axillary nodes involvement (62%) among their series. Data from the current study and other studies [10,13] reported that the histological features of BC after HL are similar to those of primary BC. Sanna et al [40] reported the same findings with the exception of the proliferation index that showed higher rates in BC among the lymphoma group as compared to the group of primary BC.

Based on concerns about possible severe consequences arising after a high total cumulative dose to the breast, several authors [6,10,13] have suggested mastectomy as the treatment of choice for BC after HL. Our case-control analysis showed that mastectomy was the predominant surgery among the lymphoma group (86%) as compared to the control group (32%). The history of previous thoracic irradiation appeared to be the reason of high rate of mastectomy in the lymphoma group, particularly if we take into account that the majority of patients had early-stage tumors. According to the difference in the surgical management, the use of adjuvant RT was significantly different between the two groups. The two patients who received RT following mastectomy did not show any radiation-related complications at their last follow-up. Nevertheless, due to paucity of data in the setting of BC after HL, the decision of RT following mastectomy should be individualized with careful outweighing of benefits and potential toxicity for each patient.

Only two patients had adjuvant RT following conservative surgery with excellent cosmetic outcome. Similarly, two studies [41,42] reported good to excellent cosmetic results, with follow-up of 30 and 46 months respectively, in 14 patients treated by lumpectomy and whole breast RT to doses of 46 to 50 Gy with 10 to 15 Gy boost to the tumor bed. Recently, Intra et al [43] presented intraoperative electron beam RT following lumpectomy as an option to avoid mastectomy in six BC patients previously irradiated for HL, but the follow-up, 30 months, is still relatively short to judge the treatment outcome. On the other hand, Wolden et al [13] reported severe soft tissue necrosis 6 years after lumpectomy and radiation (the patient was treated with tangents to 45.6 Gy and a boost of 15 Gy to the upper inner quadrant); the breast irradiation fields overlapped the prior mantle field in some regions. Overall, the small number of patients treated by a second radiation does not allow making solid conclusions, but the use of RT, and especially partial breast irradiation, warrants further investigation, particularly for women refusing mastectomy.

In the adjuvant setting, the case-control analysis showed that anthracycline-based regimens were less frequently used among the cohort of BC after HL compared to patients with primary BC. It should be noted that patients from both groups were treated at the time when the standard adjuvant CT for BC was 5'flurouracil and cyclophosphamide with either anthracylines or methotrexate. With respect to disease-free and overall survival, figures 1 and 2, respectively, show overlap of the confidence intervals indicating no significant difference between the study and the control groups at 5 and 10 years. The lack of statistical significance in presence of absolute difference of 17% in 10-year disease-free survival could be explained by the small number of patients. Furthermore, precisely because the confidence intervals on the curves are big, one should not take this difference at the face value that is the real difference could be 0% or 17% in the opposite direction. Therefore, based on our data, we could not reject the null hypothesis of similar disease-free and overall survival for the group of BC after HL and that of primary BC.

Similiary, Yahalom et al [10] reported that the prognosis of patients with BC after HL was strongly dependent on their axillay nodal status with the survival data similar to survival information of patients with primary BC. Two other studies [13,39] reported the dependence of disease-free survival for BC after HL on the disease stage exactly like the primary BC.

On the other hand, Hancock et al [3] reported that survival of BC that occurred in previously irradiated HL patients tends to be slightly lower than expected for BC in the general population. Sanna et el [40] also reported that patients with BC after HL experienced significantly lower disease-free and overall survival; they attributed these findings to the reduced use of anthracyclines in the adjuvant treatment and/or genetic damage by previous therapies and ultimately treatment resistance. We don't think that our patients with BC after HL were undertreated in terms of adjuvant therapy. The reduced use of postmastectomy RT could be explained by the fact that the majority of this group has early-stage breast cancers with negative or less than three positive axillary lymph nodes that would have been eligible for breast-conserving surgery (as shown in the control group) despite the prior irradiation that made mastectomy the preferred option of treatment. In terms of adjuvant systemic therapy, our patients with BC after HL were treated with CT and/or hormone therapy as indicated. Although we could not exclude possible resistance to CT due to prior therapy as suggested by Sanna et al [40], it might be difficult to determine if this resistance is limited to certain types of CT rather than others especially if we consider that anthracyclines were given only for two patients among the group of BC after HL. Collectively, it seems more reasonable, as shown by our data, that the survival in patients with BC after HL is rather linked to the known independent prognostic factors e.g. lymph node status and tumor size same as primary BC.

## Conclusion

BC after HL is likely to occur at a young age with a strong propensity to be bilateral. The prognosis of BC after HL appears to be similar to that of primary BC. Patients counseling, screening mammography or screening MRI and self-examination should be part of long-term surveillance protocols for this population. Mastectomy appears to be a reasonable approach in most of cases; however lumpectomy and partial breast irradiation might be an alternative worthwhile to investigate for patients who refuse mastectomy.

## Abbreviations

HL: Hodgkin's lymphoma; BC: breast cancer; CT: chemotherapy; IRB: institutional review board; RT: radiotherapy; DCIS: ductal carcinoma in-situ; ABVD: doxorubicin, bleomycin, vinblastine and dacarbazine; MOPP: nitrogen mustard, vincristine, procarbazine and prednisone; MRI: Magnetic Resonance Imaging; CI: confidence interval; STLI: subtotal lymphoid irradiation; TLI: total lymphoid irradiation; IF: involved field; LVI: lymphovascular invasion

## Competing interests

The authors declare that they have no competing interests.

## Authors' contributions

MAA, KSH and AGT were involved in the initial study conception and draft writing. MAA suggested the design of the case-control analysis. AN and SIG were involved in the statistical analysis. All authors read and approved the final manuscript.
